# Exploration of Factors Associated With Surgeon Deviation From Practice Guidelines for Management of Inguinal Hernias

**DOI:** 10.1001/jamanetworkopen.2020.23684

**Published:** 2020-11-19

**Authors:** Anne P. Ehlers, C. Ann Vitous, Anne Sales, Dana A. Telem

**Affiliations:** 1 Department of Surgery, University of Michigan, Ann Arbor; 2 Center for Healthcare Outcomes and Policy, University of Michigan, Ann Arbor; 3 Department of Learning Health Sciences, University of Michigan Medical School, Ann Arbor; 4 VA Ann Arbor Healthcare System, Ann Arbor, Michigan

## Abstract

**Importance:**

Despite availability of evidence-based guidelines for surgery, many patients receive guideline-discordant care. Reasons for this are largely unknown. For example, evidence-based guidelines recommend a minimally invasive approach for persons with bilateral or recurrent unilateral inguinal hernias. Benefits are also noted for primary unilateral inguinal hernia. However, findings from previous quantitative research indicate that only 26% of patients receive this treatment and only 42% of surgeons offer a minimally invasive approach, even for recurrent or bilateral hernias.

**Objective:**

To explore factors associated with surgeon choice of approach (minimally invasive vs open) in inguinal hernia repair as a tool to gain an understanding of guideline-discordant care.

**Design, Setting, and Participants:**

Qualitative study performed as part of a larger explanatory sequential mixed methods design. Purposive sampling was used to recruit 21 practicing surgeons from a large statewide quality collaborative who were diverse with regard to practice type, geographic location, and surgical specialty. Qualitative interviews consisted of a clinical vignette, followed by semi-structured interview questions. Through thematic analysis using qualitive data analysis software, patterns within the data were located, analyzed, and identified. All data were collected between April 24 and July 31, 2018.

**Exposure:**

Clinical vignette as part of the qualitative interviews.

**Main Outcomes and Measures:**

Capture of surgical approaches and factors motivating decision-making for inguinal hernia repair.

**Results:**

Of the 21 participating surgeons, 17 (81%) were men, 18 (86%) were White, and all were 35 years of age or older. Data revealed 3 dominant themes: surgeon preference and autonomy (eg, favoring one approach over the other), access and resources (eg, availability of robot), and patient characteristics (eg, age, comorbidities).

**Conclusions and Relevance:**

Decision-making for the approach to inguinal hernia repair is largely influenced by surgeon preference and access to resources rather than patient factors. Although a one-size-fits-all approach is not recommended, the operative approach should ideally be informed by patient factors, including hernia characteristics. Addressing surgeon preference and available resources with a clinician-facing decision aid may provide an opportunity to optimize care for patients undergoing inguinal hernia repair.

## Key Points

**Question:**

What factors are associated with choice of approach (open vs minimally invasive) for inguinal hernia repair?

**Findings:**

In this qualitative study of 21 practicing surgeons who perform abdominal wall hernia repairs, surgeon preference and autonomy, access and resources, and patient characteristics influenced the approach used for inguinal hernia repair surgery.

**Meaning:**

Addressing surgeon preference and available resources may provide an opportunity to optimize care for patients undergoing inguinal hernia repair, while understanding these motivations may also inform questions of guideline-discordant care more broadly in surgery.

## Teaser

This qualitative study explores factors associated with surgeon choice of an open vs minimally invasive approach to inguinal hernia repair to better understand deviations from guidelines.

## Introduction

Although evidence-based guidelines are increasingly available, it is well recognized that simply publishing a guideline will not achieve the desired practice change.^1,2,3^ This has been demonstrated to be true in many clinical scenarios including the prevention of spontaneous preterm birth,^4^ outpatient assessment of cardiovascular risk factors,^5^ and childhood immunization.^6^ Despite attempts to address issues related to guideline-based practice change through research and implementation efforts, adherence to guidelines remains low.^7^ Although the intention of guidelines is to improve clinical care by facilitating delivery of evidence-based therapies, lack of adherence is a significant contributor to preventable patient harm and costs the US health care system nearly $1 trillion annually.^7^

As an example, evidence-based practice guidelines recommend a tailored approach to inguinal hernia management based on the individual patient. Specifically, the guidelines recommend a minimally invasive approach to bilateral inguinal hernia or recurrent inguinal hernia that was previously repaired with open surgery.^8,9^ For bilateral inguinal hernia, this is important because both hernias can be repaired in 1 operation vs 2 operations (a staged repair) with the open approach. Despite these guidelines, only 42% of surgeons offer a minimally invasive approach to this patient population.^10^ Findings from previous quantitative research suggest that surgeon characteristics such as age, practice type, and location are largely responsible for the identified gap in guideline-discordant care.^10^ However, the nuanced reasons for deviation from practice guidelines remain poorly understood not only for inguinal hernia repair (IHR) but also for a wider range of other surgical procedures.

Using IHR as an example, we sought to explore factors associated with choice of approach to better understand deviations from guidelines. We conducted semi-structured qualitative interviews among surgeons participating in the Michigan Surgical Quality Collaborative. We aimed to elicit self-reported motivating factors for choice of approach for IHR. In addition, we aimed to describe self-reported justifications for deviation from guidelines when applicable.

## Methods

### Study Design

This study is the second component of an explanatory sequential mixed methods study^11^ that aims to understand surgeon choice of approach (eg, open vs minimally invasive) for IHR. The quantitative findings from this study have been published previously,^10^ and herein we present the qualitative component. This study was approved by the University of Michigan Medicine Institutional Review Board and followed the Consolidated Criteria for Reporting Qualitative Research (COREQ) reporting guideline.

### Interview Participants

Twenty-one surgeons participating in the Michigan Surgical Quality Collaborative were purposively included in the study sample to ensure a diverse group with regard to years in practice, practice type, and practice location (ie, maximum heterogeneity).^12^ Participants were eligible if they were practicing surgeons who performed abdominal wall hernia repairs. All participants consented to be interviewed and to have their responses published; they were not monetarily compensated for their participation in interviews.

### Interview Topic Guide

The interview guide used in this study has been previously described^13^ and is included in the eAppendix in the Supplement. The interview guide contains a variety of clinical vignettes that describe potentially controversial topics in abdominal repair and IHR as well as questions that seek to elicit explanations for variation in hernia management based on both patient and hernia factors. After 2 initial pilot interviews with participating surgeons, we made several minor changes to the interview guide. Results from the pilot interviews are included because the guide used in those interviews is similar to the final guide.

For this aspect of the study, participants were presented with a clinical vignette featuring a 24-year-old male patient with a primary unilateral inguinal hernia. The vignette was sequentially updated during the interview to vary the patient presentation (eg, recurrent hernia, bilateral hernia) and to present opportunities for surgeons to describe their rationale for choosing one approach over another. We obtained information from this vignette as well as references to IHR at other points in the interview to inform our findings.

### Data Collection

An email message outlining the purpose of the project and requesting participation in a survey designed to gather demographic characteristics was sent to each potential participant identified through the Michigan Surgical Quality Collaborative. Independent interviews were conducted by 3 members of the research team (including C.A.V.) with extensive experience in interviewing surgeons by telephone or in person. Interviews were digitally recorded and lasted between 30 and 60 minutes. All recordings were transcribed and deidentified. Observations about each interview (ie, field notes) were documented after each interview. Interviews continued until data saturation was achieved, determined when new themes emerged infrequently and the code definitions remained stable.^14^ All data were collected from April 24 through July 31, 2018. Transcripts were not returned to participants for review.

### Statistical Analysis

After data collection was complete, we iteratively analyzed the data using inductive thematic analysis. Two members of the team (A.P.E., C.A.V.) created the code book by performing a targeted review of the transcripts, searching for discussion surrounding inguinal hernia. After coming to agreement on the code book, 1 team member (A.P.E.) independently coded each transcript using NVivo Pro, version 12.6.0.959 (QST International). After the data were coded, 3 team members (A.P.E., C.A.V., and D.A.T.) reviewed the reports generated by NVivo Pro for each code to identify overarching themes.

## Results

In total, 31 surgeons completed the survey, and of those, 21 agreed to be interviewed. Among the participants, 17 (81%) were men, 18 (86%) were White, and all were 35 years of age or older. Other surgeon characteristics are given in Table 1. Reasons for nonparticipation were not elicited. We found 3 themes that characterized choice of approach for IHR: surgeon preference and autonomy, access and resources, and patient characteristics.

**Table 1.** Characteristics of the 21 Participating Surgeons

Variable	No. (%)
Sex	
Male	17 (81)
Female	4 (19)
Age, y	
35-44	9 (43)
45-54	8 (38)
55-64	3 (14)
≥65	1 (5)
Race	
White	18 (86)
Prefer not to answer	3 (14)
Ethnicity	
Non-Hispanic or non-Latino	16 (76)
Middle Eastern	1 (5)
No response given	4 (19)
Degree	
MD	14 (67)
DO	7 (33)
Duration of practice, y	
0-4	2 (10)
5-10	5 (24)
11-15	5 (24)
16-20	3 (14)
≥21	6 (29)
Completed fellowship training	
Yes	6 (29)
No	15 (71)
Fellowship concentration	
Trauma	2 (33)
Minimally invasive	3 (50)
Surgical critical care	1 (17)
Hospital demographic	
Community	8 (38)
Academic	13 (62)
Cases involving abdominal wall hernia repair, %	
11-24	14 (67)
25-49	4 (19)
50-74	3 (14)

### Surgeon Preference and Autonomy

Surgeons reported that they often selected an approach based on their preference rather than what guidelines recommend. When justifying their choice of approach, surgeons used several lines of reasoning. Some surgeons selectively referenced the evidence that best supported their approach, stating that there were data for and against both open and minimally invasive repairs and that their interpretation of these data supported their approach. Surgeons also stated that the available evidence was not of high quality and therefore the true outcome of either approach was largely unknown. Outside of guidelines or evidence, in some cases, surgeons described their personal affinity for one approach over the other. Representative quotes are included in Table 2.

**Table 2.** Representative Quotes From Participants According to Major Themes in Choice of Approach to Inguinal Hernia Repair

Theme	Quote
Preference and autonomy	“Because, I mean, I really think you can do, you can do an open approach, or you can do these robotically. My confession is, my favorite case to do right now is a robotic inguinal hernia, like I love doing them.” “Yeah, my preference really is, for a unilateral hernia, a small plug and patch repair I’ve found works really well, minimal pain, is very cost-effective.” “Yeah, so there's, you can find evidence for anything you want to do, right? So, there are always going to be people who use evidence to support their own practice.” “Well, I think a minimally invasive approach is better than an open approach even for unilateral. So then when it’s minimally invasive, then your options are either laparoscopic or robotic. And I am trained in both. I know how to do both, but I prefer the robotic approach.”
Access and resources	“I only get 2 days a month to do inguinal hernias on the robot. So, there are times where I just have to do them with a different technique because I don't have the access to the equipment. That's really not good for patient care.” “I would do a laparoscopic, if I'm at the hospital. If I'm at the surgery center, I'd typically do those open, and that's just a cost decision.” “I don’t like the laparoscopic, the stiffness of trying to close the defect with the straight sticks, so if I can’t get on the robot, then I usually just plan on doing an open repair.”
Patient characteristics	“But if you’re talking about a bilateral inguinal hernia, then you’re not doing the best operation for that patient. So, if that patient could get a laparoscopic repair, that would be the better operation for the patient. So, a surgeon should never do an inferior operation for the patient.” “I think for inguinal hernias, I'll have a discussion with a patient regarding, you know, if it's a primary repair vs a revision or re-do would change my initial approach for the inguinal hernia, depending on the way they had it. You know, if it's an initial hernia, then my usual first approach is open repair. Then if they've had a previous repair of an inguinal hernia, if it was laparoscopic, then I'd do it open.” “It depends. If he were 80 or 85, if he were healthy, he probably, he could still be done robotically. If he was unable to undergo general anesthesia, then he gets an open repair.” “The only reason I wouldn't do a laparoscopic repair is if somebody couldn't tolerate general anesthesia. So, if they had a cardiac problem, or if they had a really bad COPD, where they wouldn't be able to tolerate general anesthesia, then I would offer them an open repair, just under local. But if they can tolerate general anesthesia, then I think laparoscopic repair is a better repair and so I would do that repair.”

Abbreviation: COPD, chronic obstructive pulmonary disease.

### Access and Resources

Availability of and access to minimally invasive technologies was broadly cited as a motivating factor. In some cases, a lack of availability of resources was cited as a barrier, particularly when the robotic platform was described. For example, when the robot was not available, surgeons reverted to whichever approach they otherwise favored (eg, laparoscopy or open) rather than defaulting to the guideline-recommended approach. Cost of surgery was brought up as well, both to support the utility of the minimally invasive approach or to argue against it. Representative quotes are included in Table 2.

### Patient Characteristics

Patient characteristics such as overall health, hernia characteristics, and type of repair for recurrent hernias informed the choice of approach. In some circumstances, surgeons also mentioned patient preference as another factor that motivated their choice. Representative quotes are included in Table 2.

## Discussion

In this study, we found that the decision to offer a minimally invasive vs open approach to surgery was often appropriately motivated by patient and/or hernia characteristics. When deviation from practice guidelines was described, personal preference/autonomy and access to resources emerged as dominant themes to explain this. In circumstances in which preference and autonomy were described, surgeons stated that the available evidence to inform practice guidelines was flawed in some way, and therefore they felt empowered to use the data that best supported their practice. With regard to access and resources, surgeons often made reference to the robotic platform when determining their approach.

Previous research suggests that deviation from practice guidelines is multifactorial^2^ and that interventions to address lack of adherence should be tailored to the identified barrier.^4^ Commonly cited explanations include a lack knowledge of the guidelines,^2^ organizational or systems issues,^4,7^ maintenance of the status quo or fear of change,^4,7^ and a lack of agreement with guidelines based on personal interpretation of the data.^2^ As we found in our study, practice guidelines may also be perceived as a threat to personal autonomy.^2,15^ This is true not only for surgeons but also for physicians in general. Understanding this phenomenon allows for tailored interventions aimed at reducing deviations from practice guidelines. For example, an educational intervention to increase knowledge or awareness of practice guidelines would have little impact if the barrier to adherence were surgeon preference and autonomy.

One of the critiques of practice guidelines is that they can be somewhat ambiguous and thus open to interpretation, depending on the clinical circumstances.^7^ For example, practice guidelines for inguinal hernia management from The HerniaSurge Group (a group of international surgeons who provide hernia care) state that laparo-endoscopic repair is recommended for the repair of primary bilateral inguinal hernias provided that a surgeon with specific expertise and sufficient resources is available.^8^ One could imagine that a surgeon who only offered an open approach, or preferred the robotic platform when available but offered the open approach when it was not available, would find it to be well within the confines of the guidelines to offer a staged, open repair. Such challenges would ideally be considered by organizations when guidelines are drafted to make the recommendations as unambiguous as possible.^7^

In addition, many physicians question the evidence that was used to build guidelines in the first place, as we found in our study. Addressing this issue requires rigorous data generation to address existing gaps. For IHR, the highest-level data comparing outcomes of laparoscopic approaches with those of open approaches are limited in that studies have primarily included men and that some surgeons performing the repairs (particularly the minimally invasive repair) had variable and inconsistent experience, leading to wide differences in reported outcomes.^16,17,18^ Because there is a significant lack of outcome data for patient groups not traditionally included in these trials as well as limited outcome data from surgeons with extensive experience in each approach, the data can be called into question. Future studies should focus on addressing these gaps to strengthen the evidence to inform practice guidelines and improve the quality of care for patients.

### Limitations

This study has several limitations. First, we were unable to correlate stated practice patterns with actual practice patterns. It may be that, even among the surgeons who stated they routinely adhere to guidelines, their actual practice was different from their stated practice. For the purpose of reducing the risk that surgeons would state that they followed guidelines for social acceptability reasons, all interviews were conducted by researchers who were not surgeons. Second, we did not test knowledge of the guidelines. Therefore, surgeons who reported potential deviations from guidelines may not have been aware that they were doing so.

## Conclusions

In an ideal world, a surgeon’s decision-making would be exclusively informed by evidence-based interventions for a specific patient and not by the preference of the surgeon or whether a particular technology happened to be available that day. Although a uniform approach for all patients is not recommended, an understanding of these concepts helps to ensure that the right patient receives the right operation at the right time. Our findings from this qualitative study suggest that targeting surgeon preference and resources may provide an opportunity for increasing adherence to guidelines in surgery.

## Notes

Supplement. **eAppendix.** Interview Guide
