# Supplementary material for: Exploration of Factors Associated With Surgeon Deviation From Practice Guidelines for Management of Inguinal Hernias
Source: JAMA Netw Open. 2020 Nov 19;3(11):e2023684. doi: 10.1001/jamanetworkopen.2020.23684 (PMC7677759; doi:10.1001/jamanetworkopen.2020.23684)
Supplement: Supplement. — eAppendix. Interview Guide [file jamanetwopen-e2023684-s001.pdf]

Supplemental Online Content

Ehlers AP, Vitous CA, Sales A, Telem DA. Exploration of factors associated with surgeon deviation from practice guidelines for management of inguinal hernias. *JAMA Netw Open*. 2020;3(11):e2023684. doi:10.1001/jamanetworkopen.2020.23684

eAppendix. Interview Guide

This supplemental material has been provided by the authors to give readers additional information about their work.

© 2020 Ehlers AP et al. *JAMA Network Open.*

eAppendix. Interview Guide

Introduction
Hello, my name is [insert title, name and job identifier here]. I want to thank you for agreeing to participate in this interview. The aim of this study is to help us understand more about the surgical approach to abdominal wall hernia. As a surgeon who performs abdominal wall hernia repair, you serve as a primary source of information and will be able to provide us with valuable information that can guide evidence based hernia care. During this interview, I will ask about your behavior and perspective on how you make decisions to surgically approach various types of hernias.

This interview will take about 30 to 45 minutes. I would like to record the interview to ensure my notes are accurate. Your identity will not be disclosed to anyone outside of myself and the study team. All data will be anonymized, and after transcription, recordings of this interview will be destroyed. Identifying data will not be shared with your institution or anyone other than study investigators. You do not have to participate, you may decline to answer any questions, or stop the interview for any reason at any time.

The purpose of this conversation is to gain better understanding about how you approach hernia repair. We hope that the findings from this study will help us develop innovative strategies to develop tailored, evidence based recommendations for hernia repair.

Do you consent to participate?	Yes	No
Do you consent to audio recording of this interview?	Yes	No
Do you consent to the researchers keeping the de-identified interview data for future use about abdominal wall hernia repairs?	Yes	No
Do you have any questions or further clarification of the study and confidentiality process before we start?	Yes	No

[If consent is provided, press record.] I am now recording.

© 2020 Ehlers AP et al. *JAMA Network Open.*

Let's start with some case scenarios. I will present some example patient scenarios and ask you a few questions about how you would manage that patient and how you made your decision.

Case Scenarios:

1. A 25-year-old woman presents to your office with a symptomatic 2cm umbilical hernia with a BMI of 25 and wants elective repair.
    - How would you approach this hernia?
    - What factors motivated your decision?
    - What if her BMI was 35? 40? >50?
    - Discuss your strategy for management of hernia in women of childbearing age.

    Probe: location of hernia, recurrent versus primary hernia, timing of repair

2. An 81-year-old male presents to your office with a 5cm incisional hernia requesting elective repair.
    - How would you approach this hernia?
    - What factors motivated your decision?
    - Discuss your general strategy for management of hernia in older adults.

    Probe: location of hernia, primary hernia, recurrent incisional hernia

3. A 34-year-old woman with a BMI of 40kg/m^2 presents to your office with a symptomatic 4cm ventral hernia.
    - How would you approach this hernia?
    - What factors motivated your decision?
    - Discuss your strategy for management of hernia in the obese patient.

    Probe: location of hernia, recurrent versus primary hernia, preoperative weight management

4. A 46-year-old male with a past medical history significant for diabetes presents to your office desiring repair of a 3cm umbilical hernia. Last glycosylated hemoglobin was 11.1%. BMI is 30.
    - How would you approach this hernia?
    - What factors motivated your decision?
    - What if the BMI was >35? >40?
    - Discuss your strategy for management of hernia in patients with diabetes.

    Probe: location of hernia, recurrent versus primary hernia, preoperative glycemic control, hernia size

5. A 60-year-old male presents to your office desiring repair of a 10cm incisional hernia.
    - How would you approach this hernia?
    - What factors motivated your decision?
    - Discuss your strategy for management of hernia by the size of the fascial defect.

    Probe: location of hernia, recurrent versus primary hernia, hernia size, management of fascial defect (e.g. closure versus non-closure), mesh position

© 2020 Ehlers AP et al. *JAMA Network Open.*

6. A 52-year-old female with active tobacco use (30 pack-year history) presents to your office desiring repair of a 4cm ventral hernia.
    - How would you approach this hernia?
    - What factors motivated your decision?
    - Discuss your strategy for management of hernia in patients with active tobacco use.

    Probe: location of hernia, recurrent versus primary hernia, hernia size, smoking cessation requirements

7. A 45-year-old otherwise healthy woman presents to your office with a 0.5cm ventral hernia that is asymptomatic.
    - How would you approach this hernia?
    - What factors motivated your decision?
    - Discuss your strategy for management of hernia in asymptomatic patients.

    Probe: location of hernia, hernia size, watchful waiting criteria

8. A 24-year old presents with a primary, symptomatic unilateral inguinal hernia. Exam confirms a reducible hernia without scrotal extension. No hernia is palpated on the contralateral side.

- How would you approach this hernia?
- What factors motivated this decision?
- What if the hernia were bilateral?
- What if the hernia were recurrent?
- Patient is 85 years old, how would you approach this?
- Discuss your strategy for management of inguinal hernia.

© 2020 Ehlers AP et al. *JAMA Network Open.*

I have a few more questions that I would like to ask you about hernia repair. Before we begin, when I say MIS, I am referring to minimally invasive technique which encompasses both laparoscopic and robotic hernia repair.

1. Describe your personal experience in treating patients with hernias.
2. How many patients do you anticipate treating with minimally invasive surgery (MIS) over the next year? What about with open repair?
3. What guidelines do you follow for hernia repair? Can you describe the process of how you develop and implement those guidelines?
4. How would you describe the consistency in approaches to hernia repairs among surgeons in your practice?
5. Can you describe any formal or informal training that your received in MIS?
6. How would you describe your skill set with MIS approaches to hernia repair? What about with open repair?
7. Walk me through the steps you take in your decision to approach a hernia with minimally invasive technique versus an open technique.
   Prompts: What, if anything, does it depend on (size, patient characteristics, patient satisfaction, peer behavior, cost)?
8. In what ways, if any, do your feelings influence whether or how you use MIS?
   Prompts: If you feel anxious about a case, do you change your approach? How does your gut, previous experience, anecdotal practice influence your choices?
9. What are the main barriers to you using MIS? What about the facilitators?
   Prompts: What specific factors would help or hinder you (time in operating room, equipment)? What specific factors encourage you (feedback (outcomes, patient satisfaction), financial incentives)? Which of these helps you most?
10. In your medical opinion, what is the evidence for using MIS in hernia repair?
    Prompts: Differential outcomes by patients
11. In your medical opinion, how will treating a patient with MIS versus open surgery impact the patient in the short-term? What about the long-term?
12. In your experience, what are the perceptions of how patients feel after MIS versus open approaches?
13. In your medical opinion, what are the benefits, if any, of using MIS versus open repair?
    Prompts: to patients, colleagues, self, following guidelines, being respected by colleagues
14. What influential individuals or groups are in favor or against using MIS? Can you describe their perspective on MIS?
    Prompts: clinical leaders, colleagues, management, patients, top researchers, etc
15. How do the opinions of these people influence your decisions on whether or how you use MIS for hernia repair?
16. How important is it to you to have MIS as part of your hernia practice? [if important, close interview; if not important, proceed to 17]
17. If you sensed that not using MIS damaged your relationships in any way (with patients or providers), would this change the way that you thought about it?

© 2020 Ehlers AP et al. *JAMA Network Open.*

Conclusion:

That’s all the questions I have for you. Has anything occurred to you about this topic that I haven’t asked about?

Thank you again for your time.

© 2020 Ehlers AP et al. *JAMA Network Open.*
